# Assessment of third-year medical students’ comfort and preparedness for navigating challenging clinical scenarios with patients, peers, and supervisors

**DOI:** 10.1186/s12909-020-1984-1

**Published:** 2020-03-12

**Authors:** Anna R. Kahkoska, Tracy M. DeSelm, Laura A. Young

**Affiliations:** 1 Department of Nutrition, University of North Carolina School of Medicine, 135 Dauer Drive, Chapel Hill, NC 27599 USA; 2 Department of Emergency Medicine, University of North Carolina at Chapel Hill, Chapel Hill, NC 27599 USA; 3 Division of Endocrinology, Diabetes and Metabolism, University of North Carolina at Chapel Hill, Chapel Hill, NC 27599 USA

**Keywords:** Medical education, Diversity, Professional development

## Abstract

**Background:**

Medical training focuses heavily on clinical skills but lacks in training for navigating challenging clinical scenarios especially with regard to diversity issues. Our objective was to assess third-year medical students’ preparedness to navigate such scenarios.

**Methods:**

A 24-item survey was administered electronically to third-year medical students describing a range of specific interactions with patients, peers, and “upper-levels” or superiors including residents and attendings, spanning subjects including gender, race/ethnicity, politics, age, sexual orientation/identity, disability, and religion. Students rated their level of comfort via a 5-point Likert scale ranging from 1 (“Very Uncomfortable”) to 5 (“Very Comfortable”). Basic demographics were collected and data were summarized for trends.

**Results:**

Data were analyzed from 120 students (67% response rate, 54.2% female, 60.8% non-Hispanic white). Students reported lower comfort with peer and superiors compared to patient interactions (*p* < 0.0001). Students reported the highest comfort with sexual orientation/identity- and religion-related interactions (median (IQR): 3.3 (1.3) and 3.4 (10.0), respectively) and the lowest comfort with gender-, race/ethnicity-, and disability- related interactions (median (IQR): 2.3 (1.3), 2.0 (1.0), 2.5 (1.5), respectively). Males reported significantly higher median comfort levels for scenarios with upper-level, gender, and religion related interactions. Males were more likely to be completely comfortable versus females across the 24 scenarios, although multiple male response patterns showed evidence of a bimodal distribution.

**Conclusions:**

Third-year medical students report generally inadequate comfort with navigating complex clinical scenarios, particularly with peers and supervisors and relating to gender-, race/ethnicity-, and disability-specific conflicts. There are differences across gender with regards to median comfort and distribution of scores suggesting that there is a subgroup of males report high/very high comfort with challenging clinical scenarios. Students may benefit from enhanced training modules and personalized toolkits for navigating these scenarios.

## Background

Medical school training is heavily focused on building clinical skills, but often lacking training in navigating challenging clinical scenarios, particularly those involving diversity-related issues. Despite evidence that medical students in the United States value diversity and cultural competence among peers and superiors [1, 2], recent research indicates that overall, medical students largely struggle with issues that diversity raises in medical practice [1]. Contemporary curricula often include robust training on empathy, compassion and useful skills to address disparities and inequities in health care; missing however may be the cultivation of conflict management skills. To this end, it has been suggested that medical students would universally benefit from a curriculum that not only emphasizes clinical skill building, but also conflict management and self-reflection around diversity-related topics [1].

Although there are descriptive manuscripts on how to develop a curriculum to increase comfort with challenging clinical scenarios [3], few studies have examined how medical students trained in a traditional curriculum perceive their comfort and preparedness currently with regards to different types of interpersonal interactions and challenging scenarios stemming from differences in age, gender, religious or cultural beliefs, sexual orientation or identify, political views, and disability. Data representing interactions with patients, peers, and superiors with a broad range of diversity-related issues may inform the development of further teaching modules or case-based education to better prepare medical students to navigate these challenging clinical scenarios.

The third year of medical school marks the transition point in which students graduate from pre-clinical, classroom-based curricula to the hospital wards and clinic setting, where they experience clinical medicine for the first time and are faced with new and challenging clinical scenarios. Therefore, objective of the study was to survey third year medical students’ comfort and preparedness with a variety of diversity-related conflicts or scenarios that occur with patients, peers, and superiors spanning seven subjects, including gender, race/ethnicity, politics, age, sexual orientation/identity, disability, and religion. Based on studies showing gender-differences among medical students in empathy [4] and problem-based learning outcomes [5], as well as general gender-differences in conflict management [6, 7], we examined for differences in responses between male and female medical students.

## Methods

### Study sample

Third-year medical students at the University of North Carolina at Chapel Hill (UNC-CH) were invited to complete an electronic 24-item survey during a structured class meeting in December 2018. Medical students at UNC-CH are taught communication and conflict resolution skills through a longitudinal clinical skills training course that spans their pre-clinical years. All participants provided written informed consent, after which they were directed to an online survey. Participants received a $5 gift card upon completion. The study was approved by the Institutional Review Board at UNC-CH.

### Measures

#### Questionnaire

A 24-item survey was administered to students to assess their perceptions of comfort with different challenging clinical scenarios (see **Table,** Additional File 1, which depicts the 24 survey questions). The survey was developed with expert input to address real-life scenarios with two main considerations. First, survey items were developed to assess interactions across three “levels”, i.e. scenarios that described specific interactions with patients, peers (i.e. other medical students), and upper-levels (i.e. supervising residents and attending physicians). In addition, survey items were developed to assess interactions revolving around a total of seven subjects, or potential sources of diversity-related conflict, including gender, race/ethnicity, politics, age, sexual orientation/identity, disability, and religion. Students were asked to rate their level of comfort via a 5-point Likert scale, ranging from 1 (“Very Uncomfortable”) to 5 (“Very Comfortable”).

#### Demographic information

Upon completion of the 24 survey items, participants were asked to indicate the gender with which they identified (female, male, or prefer not to answer), the race/ethnicity with which they identified (White, Black, Mexican American, Asian Pacific Islander, Other, Mixed, or prefer not to answer), and their ethnicity (Hispanic, non-Hispanic, prefer not to answer). Race and ethnicity information were combined to give the following categories: non-Hispanic White, non-Hispanic Black, Hispanic/Mexican-American, Other/Mixed (non-Hispanic), and prefer not to answer (non-Hispanic). To reinforce inclusivity, there was an additional space for students to provide clarifying comments or provide any other information that they felt may be informative.

### Statistical analysis

Responses were de-identified and aggregated for analysis. There were no missing data. Data were summarized using median and interquartile range (IQR) overall, for each of the three levels, and each of the seven subject areas. Given evidence of non-normal distribution of composite scores, peer and superior specific comfort scores were compared to patient-specific comfort scores using Wilcoxon signed rank sum tests.

To capture possible differences in medical student comfort across gender, all analyses stratified by gender, individuals who did not indicate their gender (*n* = 2) were omitted to facilitate comparison and avoid misclassification bias associated with combining groups. A combination of summary statistics and graphical representations were used to analyze the data. Box and whisker plots and density distribution plots were constructed using the ggplot2 package in R [8]. Given the exploratory rather than deterministic nature of the pilot study analysis, *p*-values were not adjusted for multiple comparisons.

Based on trends across gender that were observed in examination of individual questions, the number of individuals who reported that they were “Completely Comfortable” (i.e. 5/5 on the Likert Scale) was calculated for 1, 4, 8. and 12 or more survey items. The proportion of males versus females were compared with a Chi squared test or Fisher’s exact test, as appropriate. Pie charts were constructed in R using ggplot2 [8].

All descriptive statistics were conducted in SAS 9.4 (SAS Institute, Cary, NC). Figures were constructed in R Version 3.4.1. *P*-values were evaluated at the 0.05 significance level and were not adjusted for multiple comparisons in the exploratory analysis.

## Results

One hundred twenty third-year medical students completed the survey, representing a response rate of approximately 67% of the total third-year medical school class. The sample was 54.2% female and 60.8% non-Hispanic white (see **Table,** Additional File 2, which illustrates the characteristics of third-year medical students who completed the Navigating Challenging Clinical Scenarios survey (*n* = 120).

Table 1 depicts student-perceived comfort scores overall, across the three levels and seven subject areas. Students reported lower comfort with peer and superior level interactions compared to patient interactions (*p* < 0.0001). Students reported the highest comfort with sexual orientation/identity- and religion-related interactions (median (IQR): 3.3 (1.3) and 3.4 (10.0), respectively) and the lowest comfort with gender-, race/ethnicity-, and disability- related interactions (median (IQR): 2.3 (1.3), 2.0 (1.0), 2.5 (1.5), respectively).

**Table 1:** Overall, Level-Specific, and Subject-Specific Comfort with Challenging Clinical Scenarios, Overall and by Gender (*n* = 118)^a^

Score, median (IQR; Q1, Q3)	All (*n* = 120)	Females (*n* = 78, 33.3%)	Males (*n* = 156, 66.7%)	*p*-value^b^
**Overall Score**^**c**^	2.8 (0.8; 2.5, 3.3)	2.7 (0.6; 2.4, 3.0)	3.0 (1.1; 2.6, 3.7)	0.025*
**Level-Specific Scenarios**^**d**^				
Patient	3.1 (0.9; 2.8, 3.7)	3.0 (0.8; 2.7; 3.4)	3.2 (1.1, 2.8, 3.9)	0.082
Peer	2.6 (1.0; 2.1, 3.1)	2.4 (0.7; 2.1 2.9)	2.7 (1.3; 2.3, 3.6)	0.123
Upper-level	2.6 (0.9; 2.3, 3.1)	2.5 (0.9; 2.0, 2.9)	3.0 (1.1; 2.5, 3.6)	0.002*
**Subject-Specific Scenarios**^**e**^				
Gender	2.3 (1.3; 1.8, 3.0)	2.0 (1.0; 1.5, 2.5)	2.8 (1.3; 2.3, 3.5)	< 0.001*
Race/ethnicity	2.0 (1.0;1.7, 2.7)	1.7 (1.3; 1.3, 2.7)	2.0 (1.7; 1.7, 3.3)	0.089
Politics	3.0 (1.2; 2.5, 3.7)	3.0 (1.0; 2.3; 3.3)	3.0 (1.3 l 2.7, 4.0)	0.526
Age	3.0 (1.3; 2.3, 3.7)	3.0 (1.3; 2.0, 3.3)	3.0 (1.7; 2.3, 4.0)	0.072
Sexual Orientation and Identity	3.3 (1.3; 2.7; 4.0)	3.0 (1.0; 2.7, 3.7)	3.7 (1.3; 2.7, 4.0)	0.065
Disability	2.5 (1.5; 1.8, 3.3)	2.3 (1.3; 1.7, 3.0)	3.0 (1.7; 2.0, 3.7)	0.097*
Religion	3.4 (1.0; 3.0, 4.0)	3.4 (1.0; 3.0, 4.0)	3.6 (1.0; 3.2, 4.2)	0.046*

Abbreviations: *IQR* interquartile range

^a^2 individuals who did not indicate gender were dropped for stratified analyses

^b^*p* values are from Mann-Whitney Test. *denotes < 0.05

^c^Scores range from 1 (“very uncomfortable”) to 5 (“very comfortable”). Overall score represents average comfort scores across all 24 scenarios

^d^Scores range from 1 (“very uncomfortable”) to 5 (“very comfortable”). Level-specific scores represent average comfort scores across scenarios that described specific interactions with patients, peers (i.e. other medical students), and upper-levels (i.e. supervising residents and attending physicians)

^e^Scores range from 1 (“very uncomfortable”) to 5 (“very comfortable”). Subject-specific scores represent average comfort scores across scenarios that described interactions revolving around a specific subject (7 total subjects including gender, race/ethnicity, politics, age, sexual orientation and identity, disability, and religion)

**Fig. 1:**
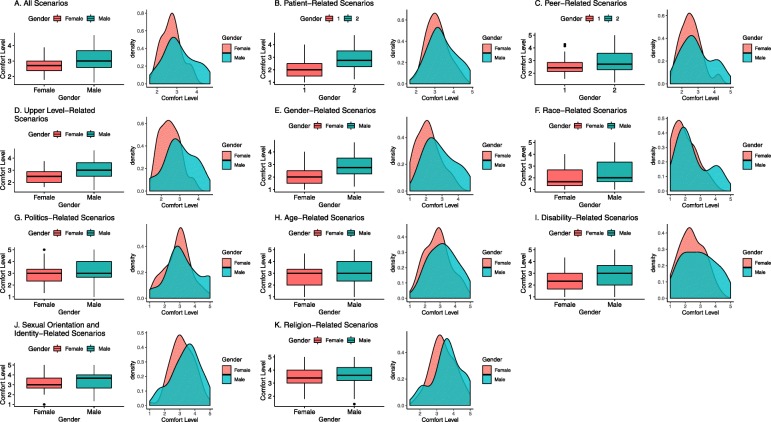
Box and Whisker and density plots for for the overall and interaction-specific challenging clinical scenarios, stratified by gender (*n* = 118). Comfort level ranges from 1 to 5, where 1 is “very uncomfortable” and 5 is “very comfortable.” For each panel, the box and whisker plot is on the left and the density plot is on the right. Panel **a** Overall score represents average comfort scores across all 24 scenarios. Panels **b**-**d** Level-specific scores represent average comfort scores across scenarios that described specific interactions with patients, peers (i.e. other medical students), and upper-levels (i.e. supervising residents and attending physicians). Panels **e**-**k** Subject-specific scores represent average comfort scores across scenarios that described interactions revolving around a specific subject (7 total subjects including gender, race/ethnicity, politics, age, sexual orientation and identity, disability, and religion)

**Fig. 2:**
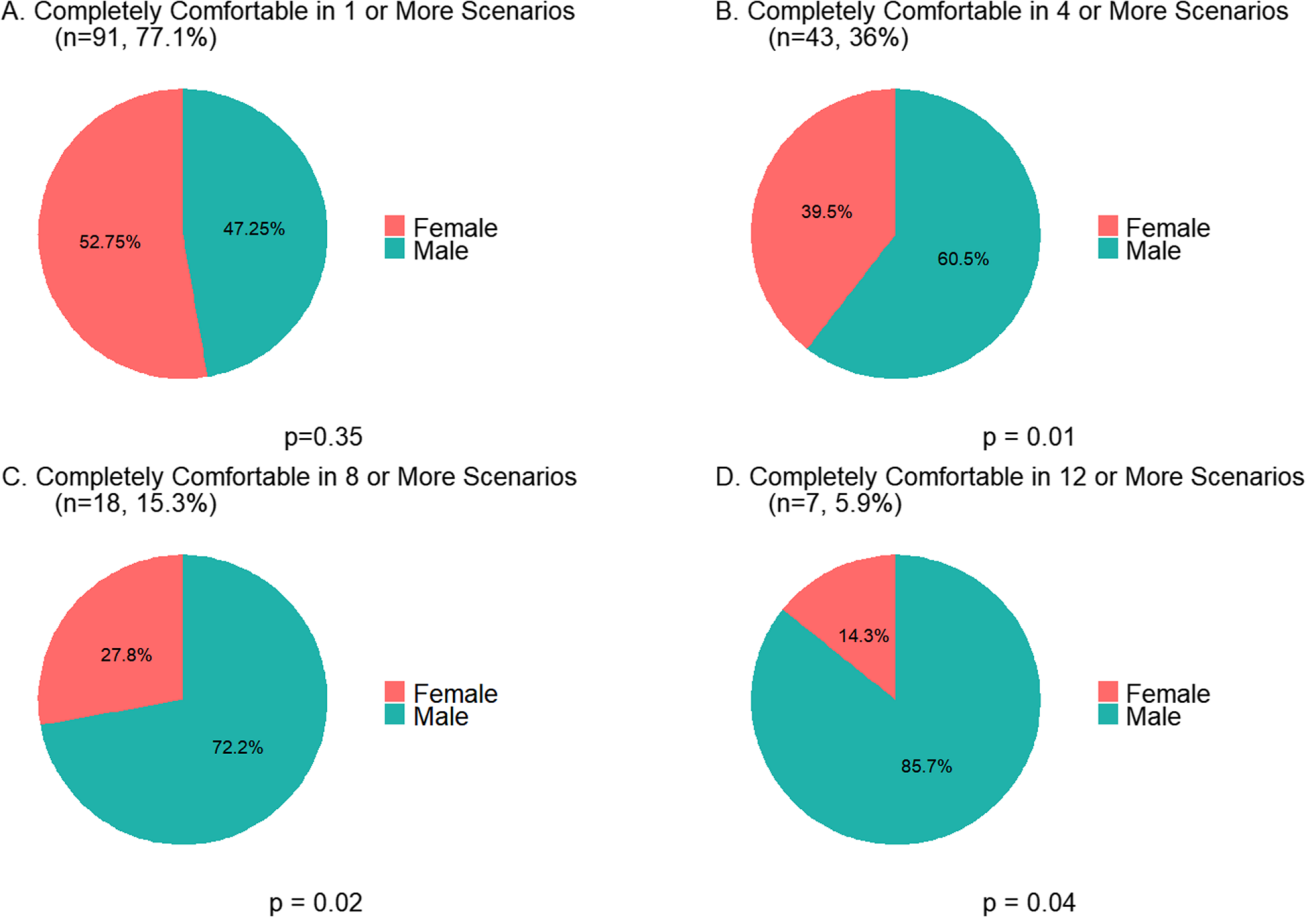
Proportion of individuals who responded as being ‘Completely Comfortable’ to different challenging clinical scenarios. Panels **a**-**d** represent the proportion who responded as being ‘Completely Comfortable’ at least 1, 4 8, and 12 of the total 24 scenarios, respectively. Results are stratified by gender. *P*-value for difference across gender is from Chi Squared or Fishers Exact test, as appropriate

There were differences across gender with regards to median comfort and distribution of scores (Table 1**,** Fig. 1). Males reported significantly higher median comfort levels for superior, gender, and religion related interactions (*p* < 0.05; Table 1). However, multiple male response patterns showed evidence of bimodal distribution that was particularly apparent in the overall score, peer-related score, superior-related score, gender-related score, and race/ethnicity-related score (Fig. 1). In addition, a higher proportion of males versus females reported being completely comfortable in 4, 8, and 12 or more of the 24 total scenarios (*p* < 0.05; Fig. 2).

## Discussion

In a pilot study including 120 third year medical students, students reported generally inadequate comfort and preparedness to navigate challenging clinical scenarios, particularly with their peers and superiors and relating to gender-, race/ethnicity-, and disability-specific conflicts. We also found significant differences across gender with regards to median comfort and distribution of scores.

A significant finding was that medical students report lower comfort with challenging clinical scenarios involving their peers and their superiors. This highlights our observation that most diversity training in medical schools focus on health care disparities and conflict with patient-specific interactions rather than peers or superiors. Conflict resolution training may occur during residency [9, 10] or for faculty and staff members [11], however to our knowledge there is a paucity of data related to teaching medical students skills in conflict management amongst peers and/or superiors. This is in stark contrast to other professional fields.

There were also significant differences across the seven subject areas, with the lowest student comfort reported around scenarios involving differences in gender, race/ethnicity, and disability.

Well documented in the literature are differences in how male and female patients are clinically evaluated and treated [12–16] how race/ethnicity disparities impact patient care [17, 18] and how disabilities can lead to suboptimal medical care [19, 20]. Unfortunately, these problematic areas relating to diversity exist outside of the patient-provider relationship to include student/superior and student peer interactions. This provides an opportunity for curricula to address the broader topic of diversity related conflict, especially in the area of gender, race/ethnicity and disability, as reflected in our data.

We found evidence of differences in responses and their distribution across gender that carry significant implications for the understanding of student needs as well as the development of future training to increase student-perceived comfort. Comparison of median scores suggests male students report higher levels of comfort overall, with superiors, and in gender- or religion-specific situations. One explanation for this finding is broader gender-differences in conflict management that have been previously proposed, where males are more likely to use forcing approach rather than other styles such as smoothing, withdrawing, or compromising, particularly with superiors [6]. However, this finding is placed into context by examination of the distribution of the responses, where a bimodal distribution was repeatedly observed with a small subset of males who report high or very high comfort level. We were able to confirm this pattern in a subsequent analysis of students who report being completely comfortable with one sixth, one third, and one half of the scenarios proposed in the questionnaires. Therefore, we are careful to avoid implying that all male students surveyed were more comfortable or prepared than the female students. Instead, data suggest that there is a subset of males who report high confidence in their abilities that skew measures of central tendency and give this illusion.

Although the construct measured in the present study was student-perceived comfort, the findings are reminiscent of a larger body of literature studying confidence in women medical trainees and leadership in the business sector. For example, a higher prevalence of imposter syndrome is well documented amongst medical students and females medical students are twice as likely to endorse symptoms compared to males [21, 22]. Studies in other context showing that the consequences of appearing confident are also different across gender [6, 7, 23]. Together, these data directly implicate the development of enhanced medical student training to address issues rooted in differences across key aspects of diversity related conflict, providing data for those areas on which to focus.

There was relatively lower comfort with peer and upper-level scenarios compared to patient-scenarios. While this may initially seem surprising; there is a continuous emphasis on conflict resolution and communication strategies focusing on the patient-provider interface during the pre-clinical and clinical years. These data highlight that students perceive a relative deficit in their abilities to navigate similar conflicts with their colleagues and superiors. Future studies integrating qualitative methodology could be particularly helpful in engaging students to articulate the type of professional training that would be most pragmatic to navigate challenging clinical situations.

The findings highlight a first step towards additional curriculum and teaching modules to address gaps in medical students’ perceived comfort and preparedness to navigate challenging clinical scenarios. Students may benefit from enhanced diversity related training modules designed to address general themes and key areas of concern identified. Additionally, a personalized teaching toolkit with an emphasis on recognition of individual vulnerabilities and biases could be developed for navigating difficult clinical situations with patients, peers, and superiors. Toolkits could include language and professional scripts, tools for de-escalation, and other interpersonal skills to promote resilience in the face of conflict.

The study should be considered in the context of its limitations. First, the single site pilot study does not address how student comfort scores may vary based on class composition or other exposure to diversity in medical school leadership. The generalizability may be affected by a demonstrated relationship between diversity in the study body and educational experiences relating to cultural competence and diversity-oriented challenges [2]. Specifically, there is a positive association between diversity of the medical student class and how comfortable students were with diversity as well as the value placed on its contribution to their medical education [24]. In addition, the response rate of 67% reflects possibly selection bias, although it not possible to analyze how non-responders to the survey may be different than those who did respond. Due to the cross-sectional nature of the pilot study, the findings cannot be used to study changes that may occur over training. Despite lacking a longitudinal aspect, the data retain value to study student perceptions of their own gaps and training needs. Finally, the Likert scale data gives limited power for discerning more nuanced differences in students’ perceived comfort. Future focus group discussions will be structured to address underlying factors or dynamics that may drive the patterns in the questionnaire data.

A further limitation of the study is the use of existing gender and race/ethnicity classifications which may not be inclusive towards all students. In particular, the gender stratified analyses employ a binary gender construct and therefore does not apply to gender identities outside the gender binary. Small sample size in the present study prohibited a more in-depth examination of how response patterns may differ across other gender identifies; however, further studies with larger sample sizes are warranted to understand how different subgroups experience challenging clinical situations and ensure that all medical students receive the training and tools from which they would benefit most.

To our knowledge, this is the first study to report data with breadth across different types of real-life challenging scenarios. This pilot research is innovative in that is considers diversity as it manifests across multiple types of relationships that affect well-being and success in medical school. The statistical analyses were carefully designed to extract all information possible from the data, including a thorough examination of distribution alongside notable response patterns. Although the final sample size may permit the use of parametric tests with Likert scale ordinal data, we used non-parametric tests to analyze the data, providing conservative estimates of significant differences [25]. These findings will directly inform the development of educational programming and tools to foster skills in navigating aspects of diversity in medical practice, as well as specific training to instill genuine confidence in students to promote a positive, diverse learning environment.

## Conclusions

We present an innovative study to assess third year medical student comfort across varying relationships (patient, peer, superior), and covering a broad range of diversity related issues. The data suggest that third year medical students report generally inadequate comfort with and preparedness to navigate complex clinical scenarios. Interestingly, the students reported lower comfort with peer and upper-level interactions compared to patient interactions, and in scenarios relating to gender-, race/ethnicity-, and disability-specific conflicts. The data also reveal differences across gender with regards to median comfort and distribution of scores, suggesting that there is a subgroup of males report high comfort with challenging clinical scenarios. Medical students may benefit from data-driven, enhanced training modules to promote the development of a personalized toolkit for navigating diversity-related issues in the workplace.

## Supplementary information

**Additional file 1 **The file depicts the 24 survey questions that were administered to third-year medical students. The mean and standard deviation (SD) and the median and interquartile range (IQR) of student scores are depicted (*n* = 120), where the range is 1 (“Very Uncomfortable”) to 5 (“Very Comfortable”).

**Additional file 2.** The file depicts the baseline characteristics of the medical students who completed the survey.

## Data Availability

The datasets used and/or analysed during the current study are available from the corresponding author on reasonable request.

## Abbreviations

IQR: Interquartile range
UNC-CH: University of North Carolina at Chapel Hill
