# Supplementary material for: Assessment of third-year medical students’ comfort and preparedness for navigating challenging clinical scenarios with patients, peers, and supervisors
Source: BMC Med Educ. 2020 Mar 12;20:71. doi: 10.1186/s12909-020-1984-1 (PMC7068976; doi:10.1186/s12909-020-1984-1)
Supplement: Supplementary file 1 — Additional file 1 The file depicts the 24 survey questions that were administered to third-year medical students. The mean and standard deviation (SD) and the median and interquartile range (IQR) of student scores are depicted (n = 120), where the range is 1 (“Very Uncomfortable”) to 5 (“Very Comfortable”). [file 12909_2020_1984_MOESM1_ESM.pdf]

**Table. Navigating Challenging Clinical Scenarios Survey Questions, with level and subject tested.** The mean and standard deviation (SD) and the median and interquartile range (IQR) of student scores are depicted (n=120), where the range is 1 (“Very Uncomfortable”) to 5 (“Very Comfortable”).				
**Level[a]**	**Subject**	**Challenging Clinical Scenario**	**Mean (SD) Score**	**Median (IQR) Score**
Patient	Political	1. You enter a room to care for a patient that has many buttons, a hat, and T shirt of a political nature that does not align with your political beliefs. She asks you who you voted for in the last election.	3.3 (1.2)	3.0 (2.0)
Upper-level	Political	2. After rounds, the discussion drifts to current political concerns. From the discussion, it becomes clear that your attending has different political views than you do. She asks you what you think about the current political situation.	2.9 (1.3)	3.0 (2.0)
Peer	Political	3. After rounds, fellow medical students on your team makes inflammatory comments about another student’s political views.	2.9 (1.1)	3.0 (1.5)
Patient	Religion	4. You enter a room wearing your traditional religious symbol (headscarf, cross, star of David, or Yarmulke, tattoo etc), and the patient asks about its meaning.	4.0 (1.0)	4.0 (2.0)
Upper-level	Religion	5. Your attending requests that the team pray with her after the loss of a patient on service.	3.6 (1.3)	4.0 (2.0)
Patient	Religion	6. You walk into a room and your patient has religiously offensive tattoos on his arms.	3.3 (11)	3.0 (1.0)
Upper-level	Religion	7. Your attending in a rural practice ask you to cover your religious accessories or tattoos, as it may offend some of his patients.	2.8 (1.3)	3.0 (2.0)
Peer	Religion	8. A patient requests that a medical school colleague pray with her; your colleague asks you to join them.	3.8 (1.2)	4.0 (2.0)
Patient	Age	9. You are asked to see a patient in the ED by your team, but the patient refuses to see such a “young, inexperienced” doctor or medical student.	3.5 (1.3)	4.0 (1.0)

Peer	Age	10. A medical school colleague requests that you see the demented elderly patient in the ED, as she "can't stand smelly, old people that can't talk to you", even though she is up for the next admission.	2.9 (1.3)	3.0 (2.0)
Upper-level	Age	11. You pull up your phone to look up a question on rounds. Your attending turns to you and says, "Put Facebook away and please join us back here on rounds."	2.5 (1.3)	2.0 (3.0)
Patient	Race/Ethnicity	12. You are sent to relay the information that your team declines to give a patient the requested narcotic prescription. The patient states that if she were another race, you would be more than happy to help her.	2.5 (1.2)	2.0 (1.0)
Peer	Race/Ethnicity	13. After an exhausting night of admissions, you overhear 2 of your student team members complaining about a difficult patient using derogatory language, including racial slurs.	2.2 (1.3)	2.0 (2.0)
Upper-level	Race/Ethnicity	14. You notice that your attending preferentially mentors and offers favoritism to other members of your team of the same race.	1.9 (1.0)	2.0 (1.5)
Patient	Gender	15. A patient of a particular religion presents with a non-emergent medical issue and requests a provider of the same sex. Due to staffing, you are unable to meet that request.	3.2 (1.1)	3.0 (2.0)
Patient	Gender	16. You are examining a patient with another medical student. The patient prefers that the 'good looking doctor' do the abdominal/GU exam.	2.3 (1.2)	2.0 (2.0)
Peer	Gender	17. A colleague is inappropriately flirtatious with the nurses to the point of being disruptive to the team and patient care.	2.2 (1.1)	2.0 (2.0)
Upper-level	Gender	18. An older attending is calling you "honey" and "dear", and occasionally resting their hand on your arm.	2.2 (1.3)	2.0 (2.0)
Patient	Sexual Orientation and Identity	19. You are asked to see a transgender patient in the ED with complications related to transitioning. The patient corrects your pronoun misuse.	4.1 (1.1)	4.0 (1.0)
Upper-level	Sexual Orientation and Identity	20. Your attending asks you to see a patient, because they believe that you are the same sexual orientation and may identify better with the patient.	3.3 (1.2)	3.0 (2.0)

Peer	Sexual Orientation and Identity	21. Prior to a clinical skills exam, a group of students are gathered and joking that a student in your group may be more familiar with the pelvic exam given their sexual orientation.	2.5 (1.2)	2.0 (1.0)
Peer	Disability	22. A student member of your team is in a wheel chair, and team is clearly getting frustrated regarding the extra time it takes to navigate the small room, and subsequent delay of rounds.	2.6 (1.2)	2.0 (2.0)
Upper-level	Disability	23. Your senior resident asks you to see a known, challenging patient in the ED with impaired hearing, using derogatory language regarding persons with disabilities.	2.4 (1.3)	2.0 (2.0)
Patient	Disability	24. You are performing a physical exam on a child with a disability. The mother is hypervigilant and continually questioning your knowledge on the patient's disease and plan.	2.9 (1.2)	3.0 (2.0)

[a]Patients refers to scenarios with inpatient and outpatient patients, peer refers to scenarios with other medical students, and upper-level refers to scenarios with residents and attending physicians.
