# Supplementary material for: Assessment of third-year medical students’ comfort and preparedness for navigating challenging clinical scenarios with patients, peers, and supervisors
Source: BMC Med Educ. 2020 Mar 12;20:71. doi: 10.1186/s12909-020-1984-1 (PMC7068976; doi:10.1186/s12909-020-1984-1)
Supplement: Supplementary file 2 — Additional file 2. The file depicts the baseline characteristics of the medical students who completed the survey. [file 12909_2020_1984_MOESM2_ESM.pdf]

**Table. Characteristics of third year medical students who completed the Navigating Challenging Clinical Scenarios survey (n=120)**	
Characteristics	N (%)
Gender[a]	
Female	65 (54.2)
Male	53 (44.2)
Prefer not to answer	2 (1.7)
Race/Ethnicity[b]	
Non-Hispanic White	73 (60.8)
Non-Hispanic Black	11 (9.2)
Hispanic or Mexican American	6 (5.0)
Asian Pacific Islander	16 (13.3)
Mixed/Other	9 (7.5)
Prefer not to answer	5 (4.2)

[a]Self-reported.
[b]Self-reported. Mixed/Other includes 2 individuals who indicated mixed race and 7 individuals who indicated other race/ethnicity.
